# Oral Administration of Alcohol-Tolerant Lactic Acid Bacteria Alleviates Blood Alcohol Concentration and Ethanol-Induced Liver Damage in Rodents

**DOI:** 10.4014/jmb.2312.12040

**Published:** 2024-01-18

**Authors:** Misun Yun, Hee Eun Jo, Namhee Kim, Hyo Kyeong Park, Young Seo Jang, Ga Hee Choi, Ha Eun Jo, Jeong Hyun Seo, Ji Ye Mok, Sang Min Park, Hak-Jong Choi

**Affiliations:** 1 Technology Innovation Research Division, World Institute of Kimchi, Gwangju 61755, Republic of Korea; 2 Pharmsville Co., Ltd., Seoul 07793, Republic of Korea; 3 Department of Biomedical Sciences, Chonnam National University Medical School, Gwangju 61469, Republic of Korea; 4 Department of Biotechnology, Graduate School, Korea University, Seoul 02841, Republic of Korea; 5 Division of Animal Science, Chonnam National University, Gwangju 61186, Republic of Korea

**Keywords:** Alcohol consumption, alcohol-induced liver damage, hangover, hepatoprotection, lactic acid bacteria, probiotics

## Abstract

Excessive alcohol consumption can have serious negative consequences on health, including addiction, liver damage, and other long-term effects. The causes of hangovers include dehydration, alcohol and alcohol metabolite toxicity, and nutrient deficiency due to absorption disorders. Additionally, alcohol consumption can slow reaction times, making it more difficult to rapidly respond to situations that require quick thinking. Exposure to a large amount of ethanol can also negatively affect a person’s righting reflex and balance. In this study, we evaluated the potential of lactic acid bacteria (LAB) to alleviate alcohol-induced effects and behavioral responses. Two LAB strains isolated from kimchi, *Levilactobacillus brevis* WiKim0168 and *Leuconostoc mesenteroides* WiKim0172, were selected for their ethanol tolerance and potential to alleviate hangover symptoms. Enzyme activity assays for alcohol dehydrogenase (ADH) and acetaldehyde dehydrogenase (ALDH) were then conducted to evaluate the role of these bacteria in alcohol metabolism. Through in vitro and in vivo studies, these strains were assessed for their ability to reduce blood alcohol concentrations and protect against alcohol-induced liver damage. The results indicated that these LAB strains possess significant ethanol tolerance and elevate ADH and ALDH activities. LAB administration remarkably reduced blood alcohol levels in rats after excessive alcohol consumption. Moreover, the LAB strains showed hepatoprotective effects and enhanced behavioral outcomes, highlighting their potential as probiotics for counteracting the adverse effects of alcohol consumption. These findings support the development of functional foods incorporating LAB strains that can mediate behavioral improvements following alcohol intake.

## Introduction

Global consumption of alcoholic beverages is an intrinsic part of many cultures and social norms. Although the deleterious effects of excessive alcohol consumption on health are well documented [1], recent attention has focused on mitigating its immediate aftermath, namely, hangovers and associated liver stress. Hangovers, characterized by headaches, nausea, and fatigue, are uncomfortable and can impair cognitive function and daily productivity [2]. In addition, repeated episodes of excessive drinking can result in long-term liver damage [3]. The search for effective interventions to alleviate these effects has led researchers to focus on functional foods. Numerous studies have consistently demonstrated that even moderate alcohol intake can lead to slower cognitive processing and delayed motor responses [4]. Such delays not only pose significant challenges in routine tasks, but can also become life-threatening in situations demanding prompt reflexes, such as driving [5]. Neuroscientific investigations have traced the neural pathways and regions most vulnerable to alcohol-induced disturbances, shedding light on the mechanisms by which alcohol dampens reaction speed [6]. Additionally, the temporal relationship between alcohol consumption and its impact on reaction time, including how long the effects persist after drinking, has been the pivotal focus of many studies [7].

Lactic acid bacteria (LAB), which are known for their probiotic properties and gut-health benefits [8], have recently been explored for their potential alcohol-tolerant capabilities. Preliminary studies suggest that certain LAB strains can metabolize alcohol and its toxic byproducts, potentially mitigating hangover symptoms [9]. Moreover, the hepatoprotective effects of these bacteria could offer a novel approach for protecting the liver from alcohol-induced oxidative stress and inflammation [10-12]. The mechanisms underlying these protective effects might be linked to the ability of LAB to modulate gut-liver axis signaling and reduce endotoxins in the bloodstream [13, 14]. Emerging evidence supports the role of alcohol-tolerant LAB as a promising functional food ingredient with the potential to alleviate hangovers and fortify liver health in the context of alcohol consumption [15].

LAB may influence neural signaling pathways by interacting with the gut microbiome, thereby altering behavioral outcomes [16]. Recent studies have documented the potential of LAB to counteract depressive-like behaviors and cognitive impairments that are often linked to alcohol consumption [17, 18]. Moreover, the protective role of LAB against the neuroinflammatory cascade commonly initiated by excessive alcohol intake underscores their therapeutic potential [19]. Some LAB strains have the ability to reduce alcohol cravings and withdrawal symptoms, paving the way for innovative interventions to treating alcohol-related disorders [20, 21]. Additionally, through the modulation of gut permeability, LAB may reduce the systemic infiltration by alcohol-derived toxins, further attenuating behavioral aberrations [22-24]. The ability of LAB to alleviate alcohol-induced behavioral anomalies may present a solution for reducing the adverse effects of alcohol [25, 26].

In the present study, we investigated the potential role of LAB in alcohol metabolism and their potential impact on blood alcohol concentration. Additionally, we investigated the effect of LAB on alleviating alcohol-induced symptoms.

## Materials and Methods

### Strains and Cell Lines

Two LAB strains, *Levilactobacillus brevis* WiKim0168 and *Leuconostoc mesenteroides* WiKim0172, were isolated from kimchi and cultured in de Man, Rogosa, and Sharpe (MRS) medium at 30°C. After examination for ethanol tolerance and potential to alleviate hangover symptoms, the selected strains were identified by sequencing the 16S rRNA gene. WiKim0168 and WiKim0172 were deposited at the Korean Culture Center of Microorganisms as KCCM 13111P and KCCM 13115P, respectively. The human liver cancer cell line, HepG2, was cultured in RPMI-1640 medium containing 10% fetal bovine serum, penicillin (100 U/ml), and streptomycin (100 U/ml). The cells were maintained in a humid incubator with 5% CO_2_ at 37°C.

### Ethanol Tolerance

LAB strains were inoculated into MRS media containing 0, 7.5, 12.5, 15, and 25% ethanol. The growth of alcohol-resistant bacteria was monitored by measuring absorbance at 600 nm using a spectrophotometer 48, 72, and 120 h post-inoculation.

### Measurement of Alcohol-Metabolizing Enzyme Activities

The alcohol dehydrogenase (ADH) and acetaldehyde dehydrogenase (ALDH) activities of WiKim0168 and WiKim0172 culture supernatants were determined using the Alcohol Dehydrogenase Activity Assay Kit (ab102533; Abcam, UK) and the Aldehyde Dehydrogenase Activity Assay Kit (ab155893; Abcam), respectively.

### In Vitro Verification of Hepatic Cell Damage Inhibition

HepG2 cells were cultured at a density of 1 × 10^4^ cells/well in 24-well plates. Following LAB treatment, cytotoxicity of the liver cells was evaluated after 24 h using CytoTox 96 Nonradioactive Cytotoxicity Assay (PR-G1780; Promega, USA).

### In Vivo Verification of Blood Alcohol Concentration Reduction and Improvement of Alcohol-Induced Liver Function

For in vivo assessment, the WiKim0168 and WiKim0172 strains were prepared by recovering the culture from a mixed medium, freeze-drying it in combination with a cryoprotectant, and storing it at 4°C as a freeze-dried powder until use. The care and study of the laboratory mice followed the protocols of the Institute Animal Care and Use Committee for the World Institute of Kimchi (WIKIM IACUC 202203). Male Sprague–Dawley rats (six weeks old; OrientBio, Republic of Korea) were fasted for 8 h, whereafter they were orally administered 25%ethanol and the LAB strains (1 × 10^10^ CFU). Blood samples were collected from rat tails at 30, 60, 180, and 300 min post-administration, centrifuged to separate the serum, and the ethanol concentration was determined using an Ethanol Assay Kit (MAK076; Sigma-Aldrich, USA). To assess liver damage, liver function was analyzed by monitoring the indicators of serum lipid metabolism using DRI-CHEM NX500 (Fujifilm Global, Japan) analyses for aspartate aminotransferase (AST) activity, total cholesterol, and triglycerides.

### Assessment of Ethanol-Related Behaviors

The alcohol-induced impairment of reflex capabilities was experimentally assessed through the loss of righting reflex (LORR) test. Male C57BL/6 mice (six weeks old) were administered ethanol (4 g/kg) to induce sleep. After intake of each LAB strain (1 × 10^9^ CFU), the time taken for the mice to awaken was measured by recording when they rolled over and straightened their limbs upwards more than three times within a minute. Onset and total sleep times were calculated to evaluate the sleep arousal efficacy of LAB.

### Evaluation of Probiotic Properties

To assess acid resistance, each LAB strain was inoculated into sterilized MRS broth adjusted to pH 2.5 using 1 N HCl and then cultured at 37°C. After incubation for 3 h, samples were plated onto MRS agar plates, which were subsequently incubated at 37°C for 24 h, and colony counts were performed to determine cell numbers. For bile salt resistance, each LAB strain was inoculated into MRS broth containing 0.3% bile salts and cultured at 37°C for 2 h. After the incubation period, samples were plated onto MRS agar plates, which were then incubated at 37°C for 24 h. Colony counts were performed to measure cell numbers. The intestinal cell adhesion ability was assessed by adding each LAB strain onto HT-29 cells cultured in 6-well plates and incubating them at 37°C for 2 h. Following incubation, non-adherent LAB were removed by washing five times with phosphate-buffered saline. Subsequently, adherent LAB were plated onto MRS agar plates, incubated at 37°C for 24 h, and colonies were counted to calculate cell number.

### Statistical Analysis

One-way and two-way analysis of variance were performed using GraphPad Prism software (version 7.05; GraphPad Software Inc., USA). All data are presented as the mean ± SEM. Statistical significance was set at *p* < 0.05.

## Results

### Alcohol Tolerance Efficacy of LAB

**Fig. 1:**
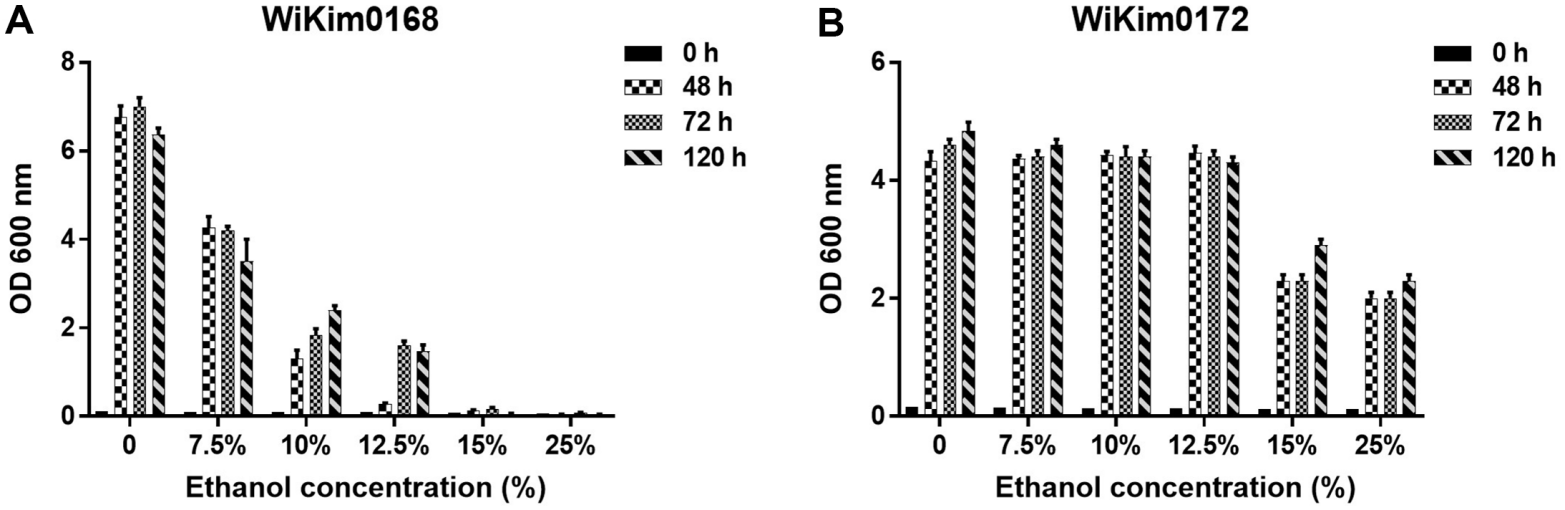
Growth of the *Levilactobacillus brevis* WiKim0168 (**A**) and *Leuconostoc mesenteroides* WiKim0172 (**B**) in MRS medium containing 7.5, 10, 12.5, 15, and 25% ethanol (v/v), as determined through absorbance measurements at 600 nm for the indicated times.

To assess the alcohol tolerance of WiKim0168 and WiKim0172, the growth of each strain was observed in MRS medium containing various concentrations of alcohol. WiKim0168 exhibited stable growth in the presence of 12.5% alcohol and delayed growth after exposure to 15% alcohol (Fig. 1A). WiKim0172 displayed consistent growth in medium with a 25% alcohol content and was identified as an alcohol-tolerant LAB strain (Fig. 1B). These findings show that WiKim0168 exhibits remarkable stability and growth in the presence of alcohol even at high concentrations, and WiKim0172 shows excellent resistance to alcohol, further highlighting its potential as a candidate for alleviating alcohol-related hangovers.

### Alcohol Dehydrogenase and Acetaldehyde Dehydrogenase Activities of Alcohol-Tolerant LAB

**Fig. 2:**
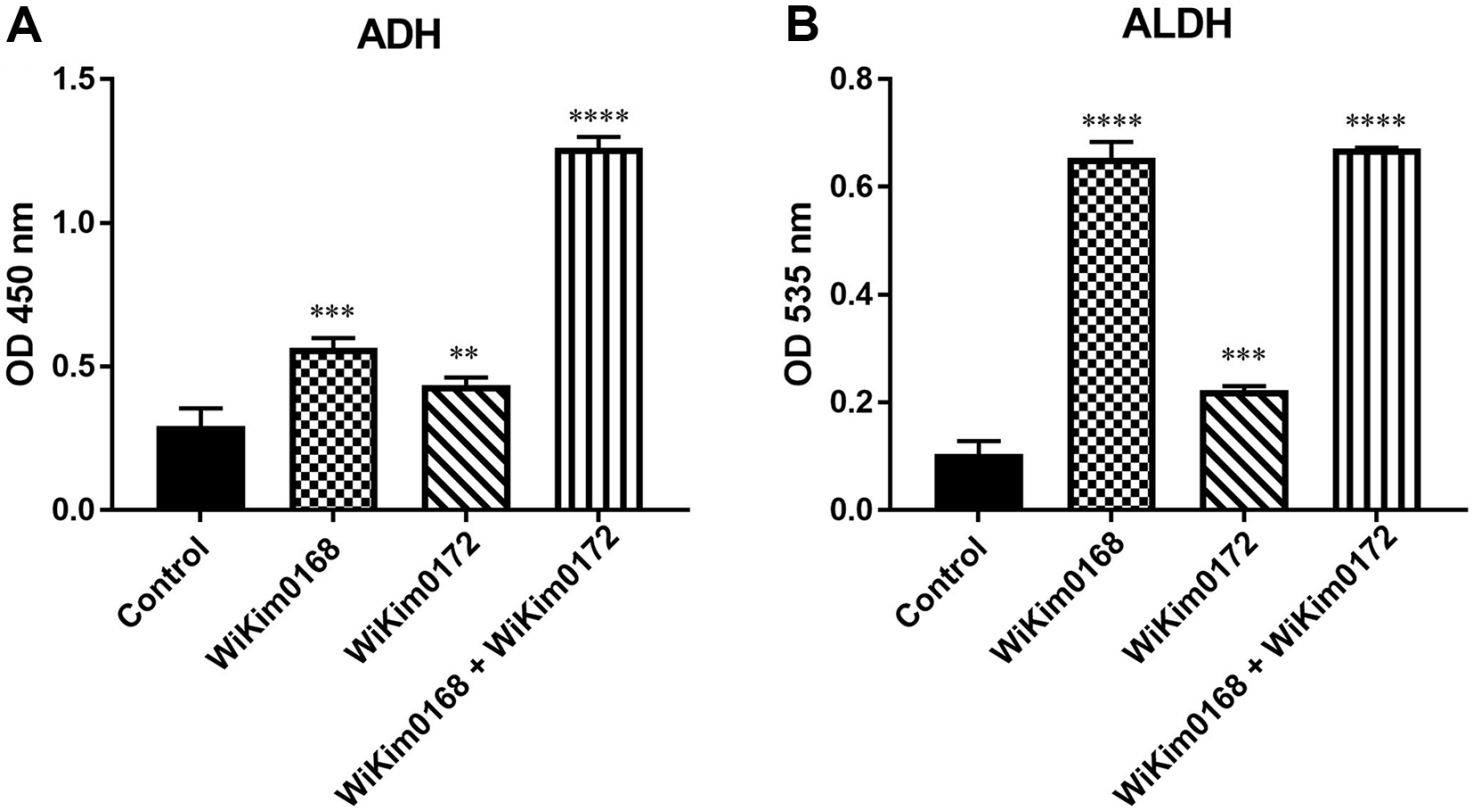
Alcohol dehydrogenase (ADH) and aldehyde dehydrogenase (ALDH) activities of *Levilactobacillus brevis* WiKim0168, *Leuconostoc mesenteroides* WiKim0172 and combination of WiKim0168 and WiKim0172. Endogenous ADH and ALDH activities shown for samples cultured for 48 h, as measured via a substrate-dependent increase in NADH absorbance. Significant differences are shown at **p < 0.01, ***p < 0.001, and ****p < 0.0001, compared with the control.

Alcohol metabolism primarily occurs in the liver via the enzymatic action of ADH and ALDH [27]. When inefficiently metabolized, alcohol accumulates in the liver, leading to the onset of various hangover symptoms. In this study, we therefore evaluated the ADH and ALDH activities of WiKim0168 and WiKim0172. WiKim0168 exhibited ADH and ALDH activities that were more than 190% and 600% higher than those of the control group, respectively (Fig. 2A and 2B). Similarly, WiKim0172 displayed 140% and more than 210% higher ADH and ALDH activities, respectively, than those of the control group (Fig. 2A and 2B). Upon combining the strains WiKim0168 and WiKim0172 in equal ratios, we noted that the enzymatic activities of ADH and ALDH exceeded those observed when the strains were utilized independently (Fig. 2A and 2B). These results suggest that these two LAB strains possess remarkable enzymatic capabilities that are crucial for alcohol metabolism and detoxification.

### Inhibitory Effect on Hepatocyte Damage by Alcohol-Tolerant LAB

**Fig. 3:**
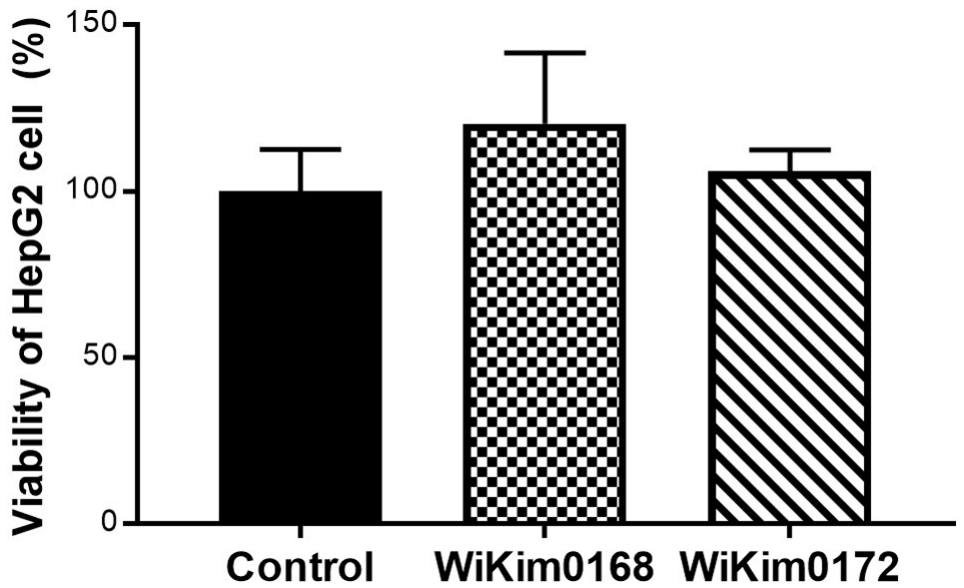
Effect of *Levilactobacillus brevis* WiKim0168 and *Leuconostoc mesenteroides* WiKim0172 on the growth of hepatocellular carcinoma cells. HepG2 cells incubated with WiKim0168 and WiKim0172 for 24 h. Cell viability was determined using the cytotoxicity assay and presented as the percentage of cell survival. Data are representative of three independent experiments and shown as the mean ± SD (*n* = 3).

To evaluate the effect of LAB on hepatocyte viability, HepG2 and each LAB strain were co-cultured, and the level of lactate dehydrogenase (LDH) enzyme secreted during cell damage was measured (Fig. 3). Co-culturing of hepatocytes with WiKim0168 resulted in approximately 20% higher cell growth in the experimental group than in the control group. In addition, evaluation of LDH release by WiKim0172 revealed a 6.3% increase in hepatocyte growth, similar to that observed in the control group. These results indicate the potential of LAB to inhibit hepatocyte damage.

### In Vivo Efficacy of Alcohol-Tolerant LAB in Alcohol Reduction

**Fig. 4:**
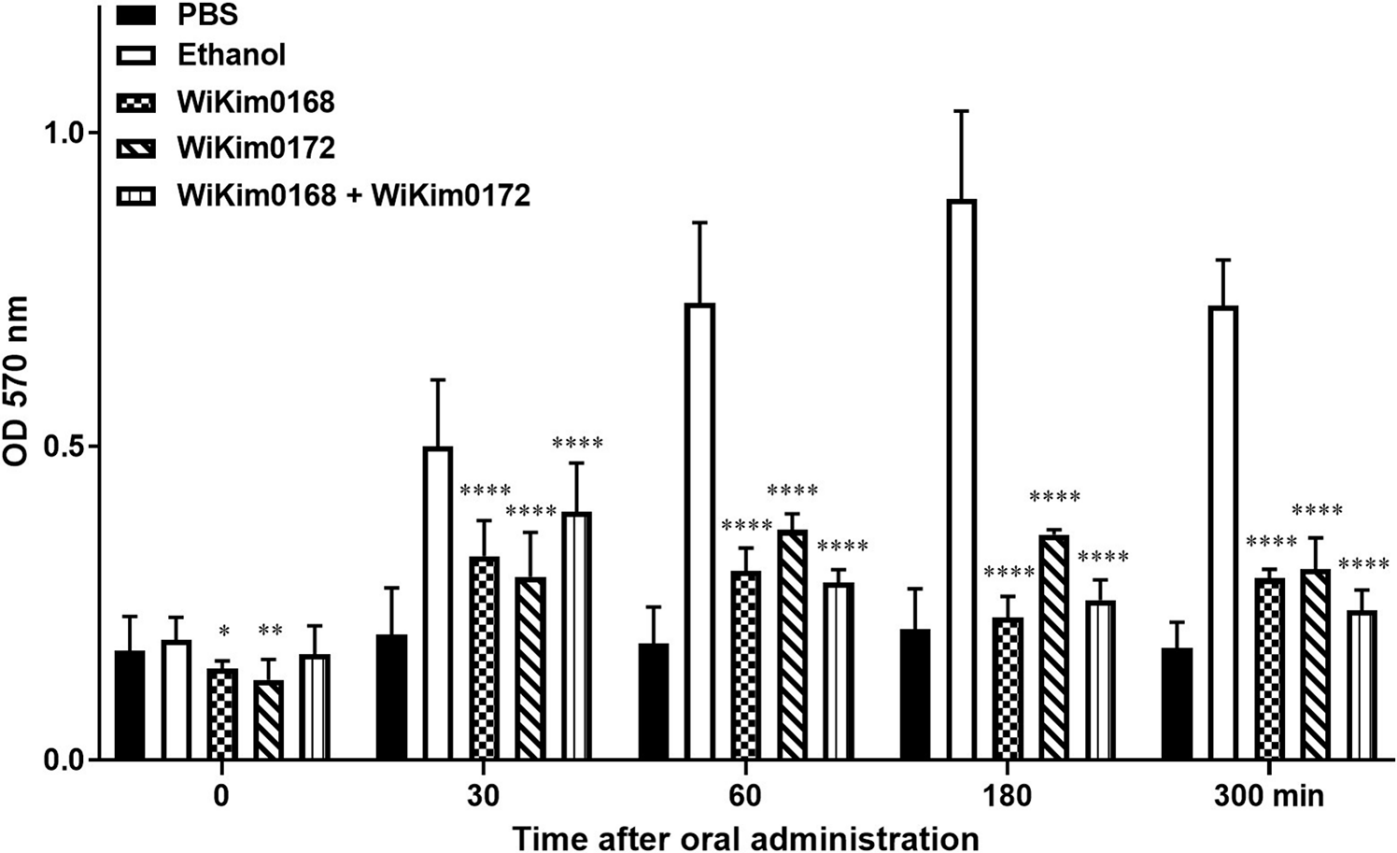
Blood ethanol concentration in the serum of rats administered with *Levilactobacillus brevis* WiKim0168 and *Leuconostoc mesenteroides* WiKim0172 after acute ethanol exposure. Significant changes in WiKim0168 and WiKim0172 feeding after the indicated times. Tail blood samples were collected at 0, 30, 60, 180, and 300 min after administration. Significant differences are shown at *p < 0.05, **p < 0.01, and ****p < 0.0001, compared with an ethanolonly control. PBS, phosphate-buffered saline.

Each WiKim0168 and WiKim0172 strain was simultaneously administered to animals with 25% ethanol, and blood samples were collected at various time points to measure and verify their alcohol-reducing efficacy. WiKim0168 exhibited a remarkable reduction in blood alcohol concentration of over 50% compared with that in the control group that received only ethanol at 60 min post-administration (Fig. 4). Similarly, WiKim0172 demonstrated a 45% and 55% reduction in blood alcohol concentration 60 and 180 min after administration, respectively, compared with that in the control group (Fig. 4). When the WiKim0168 and WiKim0172 strains were administered in combination, a substantial 60% reduction in blood alcohol concentration was observed 60 min after administration, along with an approximately 69% and over 60% reduction 180 and 300 min after administration, respectively, compared with that in the control group (Fig. 4). These results suggest the alcohol-reducing efficacy of WiKim0168 and WiKim0172, either individually or in combination.

### Improvement of Alcohol-Induced Liver Function by Alcohol-Reducing LAB

**Fig. 5:**
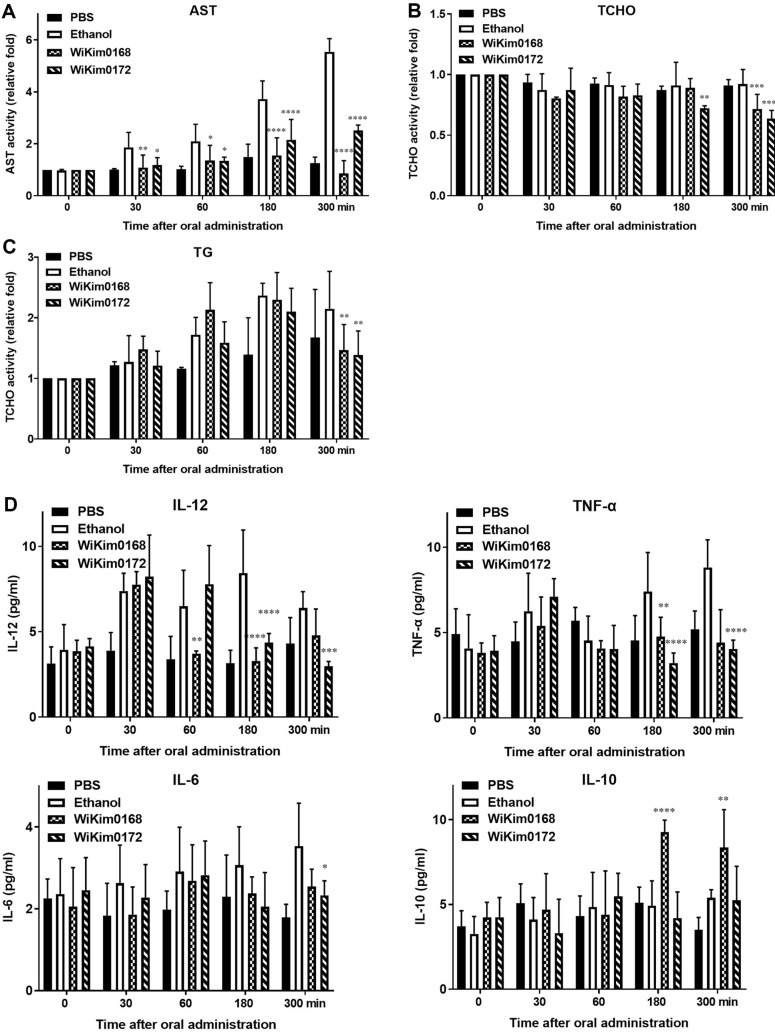
Effect of *Levilactobacillus brevis* WiKim0168 and *Leuconostoc mesenteroides* WiKim0172 on ethanol metabolism following acute ethanol exposure in rats. Data are displayed as the mean ± SD (*n* = 6). (**A**) Aspartate aminotransferase (AST), (**B**) total cholesterol (TCHO), (**C**) triglycerides (TG) and (**D**) inflammatory cytokine (IL-12, TNF-α, IL-6, and IL-10) activities at 30-, 60-, 180-, and 300-min time points. Significant differences are shown at *p < 0.05, **p < 0.01, ***p < 0.001, and ****p < 0.0001, compared with an ethanol-only control. PBS, phosphate-buffered saline.

Alcohol consumption can lead to liver damage and dysfunction, as evidenced by elevated AST levels, disturbances in lipid metabolism, and increased inflammatory cytokine activity [28, 29]. We investigated the efficacy of alcohol-reducing LAB strains in improving alcohol-induced liver function in an animal model of excessive alcohol consumption. Serum samples from these animals were analyzed to assess various parameters related to liver health, including AST activity, lipid metabolism markers (triglycerides and total cholesterol), and inflammatory cytokine-mediated activity associated with liver damage, such as the infiltration of inflammatory cells. Following the administration of alcohol-reducing LAB, significant decreases in blood AST concentration and levels of total blood cholesterol and triglyceride were observed in the WiKim0168 and WiKim0172 intake groups compared to the control group (Fig. 5A-5C). Furthermore, the secretion of pro-inflammatory cytokines such as IL-12, TNF-a, and IL-6 were reduced by LAB administration, indicating a reduction in inflammation associated with alcohol consumption. In contrast, the release of IL-10, an anti-inflammatory cytokine, was increased, suggesting that LAB might suppress systemic inflammation (Fig. 5D). These results indicate that alcohol-reducing LAB have the potential to improve alcohol-induced liver dysfunction and inflammation.

### Improvement of Awakening Efficacy from Alcohol-Induced Sleepiness (Hangover Behavior)

**Fig. 6:**
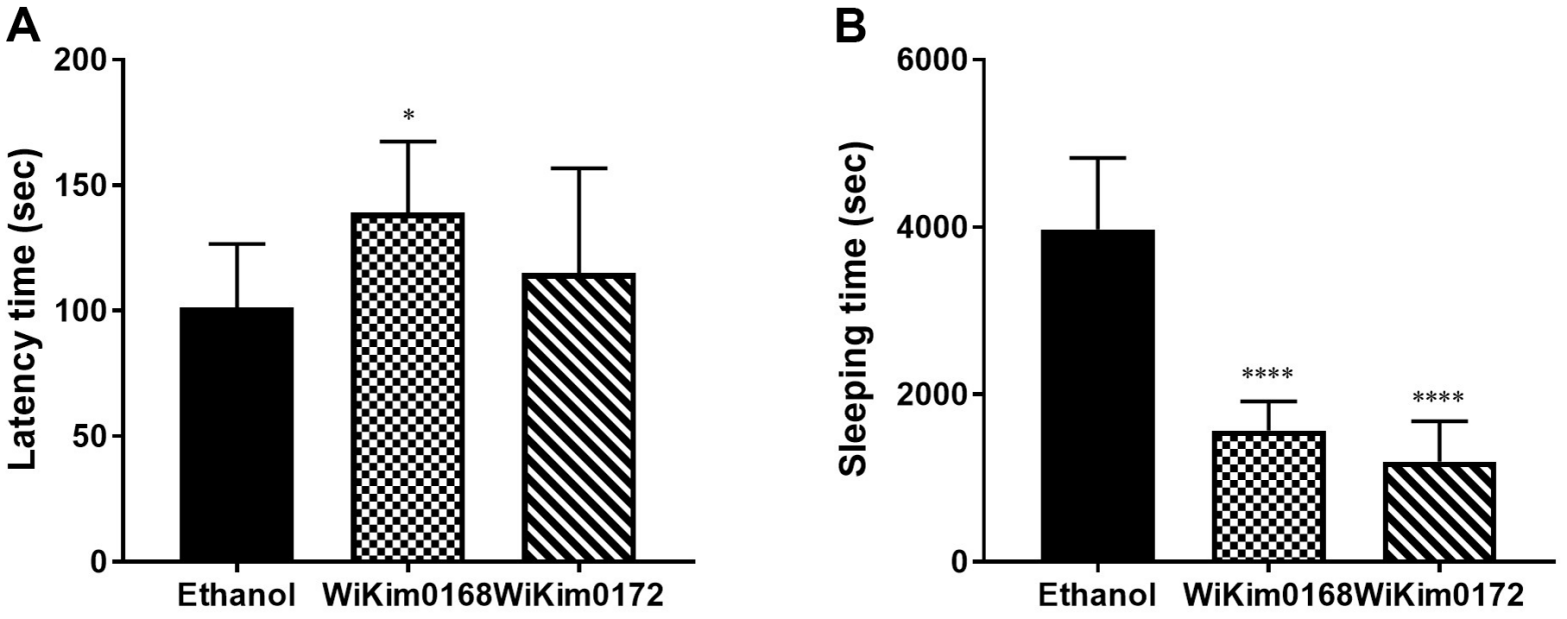
Loss of righting reflex in rodents used for assessing the sleep effects of *Levilactobacillus brevis* WiKim0168 and *Leuconostoc mesenteroides* WiKim0172. Time to sleep latency (**A**) and total sleep time (**B**) were recorded. Mice received one intraperitoneal injection of ethanol and an oral administration of each strain and were immediately placed in a supine position to check their ability to right themselves, *i.e.*, the ability to turn back over onto all four legs within 1 min. Values are represented as the means ± SD (*n* = 10). Significant differences are shown at *p < 0.05 and ****p < 0.0001, compared with an ethanol-only control.

To evaluate the efficacy of awakening from sleep induced by alcohol, we conducted the LORR test following oral administration of WiKim0168 and WiKim0172 in mice, where both onset time and total sleep duration were calculated. When WiKim0168 was administered with alcohol, it was observed that the onset time was delayed by approximately 25% and sleep awakening was approximately 45% faster compared with those in the control group given only alcohol, indicating that WiKim0168 significantly delayed the onset of alcohol-induced sleep and expedited the awakening process (Fig. 6A). Similarly, WiKim0172 intake showed delayed onset time by approximately 18% and sleep awakening approximately 50% faster compared to the control group (Fig. 6B). These findings suggest that both WiKim0168 and WiKim0172 have a positive impact on awakening from alcohol-induced sleep, with WiKim0172 exhibiting a higher efficacy in terms of reducing sleep onset time.

### Probiotic Properties of WiKim0168 and WiKim0172

**Fig. 7:**
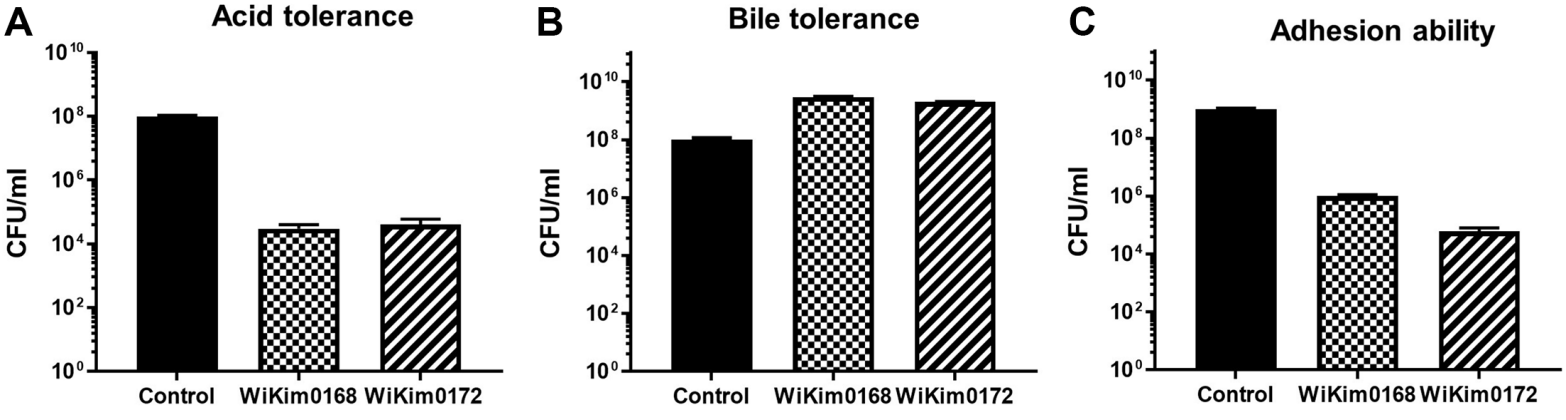
Survivability of *Levilactobacillus brevis* WiKim0168 and *Leuconostoc mesenteroides* WiKim0172 to gastrointestinal tract-related stresses, such as (**A**) gastric acid tolerance (pH 2), (**B**) 0.3% bile salt tolerance, and (**C**) adherence to HT-29 cells.

The effectiveness of probiotics largely depends on their ability to survive harsh conditions, such as the low pH environment of the stomach and the presence of bile salts in the intestines. Additionally, their capacity to adhere to intestinal cells is crucial for exerting their beneficial effects. Therefore, we evaluated the probiotic properties of both WiKim0168 and WiKim0172 strains. The results showed that WiKim0168 was able to recover to a concentration of 1 × 10^4^ CFU/ml after incubation at pH 2.5 for 3 h, exhibiting acid resistance (Fig. 7A). When it was incubated in the presence of bile salt for 6 h, a recovery rate of 3 × 10^9^ CFU/ml was obtained (Fig. 7B). Intestinal stability of WiKim0168 was observed with a recovery of 1 × 10^6^ CFU/ml after incubation with HT-29 cells for 2 h (Fig. 7C). WiKim0172 also displayed acid resistance, and could be recovered at a concentration of 4 × 10^4^ CFU/ml after incubation at pH 2.5 for 3 h (Fig. 7A). Similarly, its bile salt resistance was comparable, with a recovery rate of 2 × 10^9^ CFU/ml after incubation for 6 h (Fig. 7B). Intestinal stability of WiKim0172 was verified, with a recovery rate of 4 × 10^4^ CFU/ml after co-culture with intestinal epithelial cells for 2 h (Fig. 7C). These results indicate that both WiKim0168 and WiKim0172 exhibit probiotic characteristics such as acid resistance, bile salt resistance, and intestinal stability, thereby implicating these two strains as probiotic candidates.

## Discussion

The probiotic revolution has opened numerous therapeutic avenues for addressing various health concerns. One area that has recently gained significant momentum is the exploration of the interactions between alcohol and probiotics. Moreover, recent studies have revealed the potential role of probiotics in influencing alcohol metabolism and alcohol-induced behavior, making this a promising area for further investigation. Therefore, we examined the potential of LAB isolated from kimchi to reduce blood alcohol concentrations and mitigate the behavioral impacts associated with alcohol consumption.

In the present study, we first assessed the alcohol tolerance of LAB strains and selected WiKim0168 and WiKim0172, which exhibit high alcohol tolerance. In particular, the growth of WiKim0168 was observed even at 25% alcohol concentration. Similarly, it has been reported that several strains of lactic acid bacteria isolated from beer and cheese are able to grow in high concentrations of alcohol (up to 25% alcohol), which is closely correlated with the high expression frequency of genes involved in exopolysaccharide production [30]. We next evaluated the ADH and ALDH activities of WiKim0168 and WiKim0172 and observed that intake of LAB strains significantly enhanced ADH and ALDH activities.

Certain probiotic strains can alter the rate or extent of alcohol absorption in the intestines, potentially leading to a reduction in its concentration in the bloodstream [31]. Gut microbiota can significantly affect alcohol metabolism, with specific strains exhibiting the ability to metabolize alcohol into various metabolic products, which may, in turn, influence its systemic effects [32]. Therefore, we questioned whether administration of WiKim0168 and WiKim0172, which possess high intestinal epithelial cell adhesion capacity, could reduce alcohol concentration in the bloodstream. Our in vivo results clearly showed that intake of each LAB strain significantly reduced blood alcohol concentration, and an even higher reduction was observed in the experimental group that administered WiKim0168 and WiKim0172 in combination.

Chronic alcohol consumption causes neuroinflammation [25]. Probiotics can potentially modulate immune responses and may play a role in reducing inflammation-induced behavioral changes. Considering the pivotal role of gut microbiota in liver health, probiotics have been explored for their potential to protect the liver against alcohol-induced damage, which could indirectly impact alcohol-related behaviors. In this study, intake of LAB strains not only significantly reduced the secretion of pro-inflammatory cytokines, which is induced by liver damage, but also increased the production of the anti-inflammatory cytokine IL-10, indicating that these two LAB strains might suppress systemic inflammation. Moreover, WiKim0168 and WiKim0172 were able to significantly delay the onset of alcohol-induced sleep and promote the awakening process, demonstrating that LAB strains effectively alleviate hangover behavior.

It is worth noting that different probiotic strains may offer varying effects, underscoring the importance of selecting the appropriate strain for achieving specific therapeutic outcomes. Determining the effective dose, delivery method, and regimen for probiotic administration in the context of alcohol consumption remains an active area of investigation. The emerging understanding of the role of probiotics in modulating alcohol-related physiological and behavioral outcomes highlights the potential for harnessing LAB for therapeutic interventions. However, to translate these findings into practical applications, a more comprehensive understanding of the underlying mechanisms and conducting well-designed clinical trials are essential.

In conclusion, oral administration of WiKim0168 and WiKim0172, alcohol-resistant strains isolated from kimchi, exhibited high levels of ADH and ALDH activities. These strains effectively reduced blood alcohol levels and ameliorated alcohol-induced behavioral changes. Further research and clinical trials are required to elucidate the full potential and mechanisms underlying these effects, ultimately enabling the development of functional food products or effective therapeutic interventions.
